# Organisational Policies and Practices for the Inclusion of Vulnerable Workers: A Scoping Review of the Employer’s Perspective

**DOI:** 10.1007/s10926-022-10067-2

**Published:** 2022-09-09

**Authors:** A. Kersten, M. van Woerkom, G. A. Geuskens, R. W. B. Blonk

**Affiliations:** 1 Department of Human Resource Studies, School of Social and Behavioral Sciences, Tilburg University, Warandelaan 2, 5037 AB Tilburg, The Netherlands; 2 Healthy Living, Netherlands Organisation for Applied Scientific Research (TNO), Leiden, The Netherlands; 3 Department of Psychology, Education and Child Studies, Center of Excellence for Positive Organisational Psychology, Erasmus University Rotterdam, Rotterdam, The Netherlands; 4 Optentia Research Focus Area, North-West University, Vanderbijlpark, South Africa

**Keywords:** Inclusion, Employer engagement, Vulnerable workers, Disability, Migrant worker, Low-educated worker, Long-term unemployed, Scoping review

## Abstract

*Purpose* Current models of inclusive workplaces are primarily based on the perceptions of vulnerable workers, whereas attention for employer’s perceptions is lacking. This scoping review addresses this issue by mapping the literature that covers employer’s perceptions on the application and importance of organisational policies and practices aimed at the inclusion of vulnerable workers. *Methods* A literature search for qualitative and quantitative research articles was conducted in MEDLINE, Scopus, ProQuest, PsychInfo, Google Scholar and Web of Science. Studies were included when (a) they reported on practices aimed at the inclusion, participation, or rehabilitation of (b) workers with disabilities, a low education or migration background, or who were long-term unemployed, and (c) were based on samples of employers or their representatives. *Results* The search resulted in 3,134 articles. In total, 38 articles met the inclusion criteria of this study. We identified seven types of inclusive practices to stimulate the inclusion of vulnerable workers that employers applied and/or perceived as valuable: senior management commitment, recruitment and selection, performance management and development practices, job accommodations and redesign of work, supportive culture, external collaborations with other employers, and monitoring. *Conclusions* Our review identified seven categories of inclusive practices that pertain to all stages of the employee journey of vulnerable workers. These categories move beyond those reported in studies based on employee samples, for instance by highlighting the importance of monitoring and collaborations with other employers. Hence, our findings stress that insight into employers’ perceptions about effective measures is crucial to increase labour market participation of vulnerable groups.

**Supplementary Information:**

The online version contains supplementary material available at 10.1007/s10926-022-10067-2.

## Introduction

The number of workers facing difficulties on the labour market in terms of obtaining and maintaining a job has been increasing over the years [1]. These so-called vulnerable workers are disproportionately impacted by economic and labour market trends, such as financial crises and globalisation [2, 3], and are at the highest risk of long-term unemployment [1, 4]. Vulnerable workers can be defined as individuals, who have a high probability to end up in precarious working conditions due to the adversities they face because of for instance, a disability, a migration background, and/or limited work experience. Such precariousness is defined as “accumulated adversities”, such as low access to training and career opportunities, increased risk of job loss, or low income [5, p. 552]. Due to the recent COVID-19 crisis, the adversities that vulnerable groups face on the labour market have only worsened, and the unemployment gap between vulnerable and non-vulnerable groups continues to grow [6]. Examples of important vulnerable groups, which are the focus of this paper, are *disabled persons*, *persons with a migration background, long-term unemployed persons, and low-educated persons.*

*Disabled persons* (i.e., persons with an intellectual, psychiatric, neurological, physical, visual, or hearing disability) do not receive equal opportunities in Europe, the USA, Asia and the Pacific due to their need for adaptations in work in terms of content, place or time [7–9]. Factors that hamper disabled persons to integrate in the labour market include employers’ prejudice about their capabilities, a lack of support networks or self-esteem, inadequate transportation means, low educational attainment, and lacking training and development opportunities [10, 11]. *Migrant workers* (i.e., individuals moving to a different country or area to pursue employment, such as temporary foreign workers) also suffer severely from barriers to enter and maintain a position on the labour market in Europe, the Middle East, and the USA. They are often hired for precarious work, segregated from non-migrant employees [7, 12, 13]. The main challenges for migrant workers relate to employers’ prejudice about foreigners, xenophobia, language gaps, lack of recognition for (educational) qualifications from different countries, and legal restrictions for foreign citizens to be hired [14, 15]. *Long-term unemployed persons* (i.e., persons who have been unemployed for more than one year, such as persons in welfare) report negative experiences with successful reemployment in Europe and the USA, due to a person-job misfit [16, 17]. Among long-term unemployed workers, the return to work is hampered by a decreased ability to work due to health problems, a lack of work experience, depleted networks, negative employer attitudes, lower education levels, inadequate support in finding and keeping a job, and financial problems [18, 19]. Lastly, *low-educated workers* suffer detrimental consequences in terms of employment prospects in the global knowledge-based economy as the investments in their human capital, e.g., through training and education, both before and during their career is low [20]. Common challenges for sustainable employability are discrimination in selection procedures because of lacking formal education, learning barriers, having no professional license, and lacking language proficiency [21, 22].

Individuals may be categorised into more than one vulnerable group, e.g., a person with a disability may be long-term unemployed. And, although previous research highlights the importance of tailored practices to address (the challenges of) each of these vulnerable groups specifically e.g., [23, 24], the groups also seem to have overlapping vulnerabilities. All of these groups face employers’ biases on their capacity to work within an organisation, e.g., due to functional limitations, lacking education or experience, or language barriers. All groups also require some form of adaptations or investments by the employer to become or remain sustainable employable, e.g., a (language) training program or an adapted desk.

To promote the involvement and investments of employers in the inclusion of vulnerable workers and to reduce persisting unemployment, active labour market policies (ALMPs), such as job-search programs or unemployment insurances, have been launched [25, 26]. Even though these activation policies rely on the active involvement of employers for their effectiveness, there has been a lack of attention for employers’ roles in the workplace inclusion of vulnerable groups. However, employers have a key role in increasing inclusive workplaces. Inclusive workplaces can be defined as workplaces, in which “people of all identities and many styles can be fully themselves while also contributing to the larger collective, as valued and full members” [27, p. 235]. In order to explore the role of employers in enhancing workplace inclusion of vulnerable workers, several inclusive workplace models have been proposed that focus specifically on organisational policies and practices for inclusion. A recent example is the model by Shore et al. [26], which posits that employers can contribute to perceived inclusion and retention of minorities by implementing organisational practices. Examples of such practices are recruitment practices, diversity training, practices aimed at managing discrimination, retention practices and development practices. All these practices address the needs of vulnerable groups by both enhancing positive factors such as psychological safety and involvement, and preventing undesired factors, such as discrimination or biases. In addition, Shore et al. [26] propose a key role for senior management commitment in the inclusion of vulnerable groups. Another recent model by Jansen et al. [28] indicated that the role of employers in supporting work participation of persons with disabilities is primarily based on practices relating to offering accommodations or supervisor support.

Although these and similar studies provide valuable insights into what employers can do to stimulate labour market inclusion of vulnerable groups, most of the literature is based on e*mployees’* perspectives [3, 29, 30]. Insights into key organisational practices, as seen from the viewpoints of *employers*, are lacking [31–34]. This is an important omission, since employee and employer perceptions of organisational practices may differ, and not all organisational practices may be directly perceived by employees [29]. Furthermore, the strategic decisions of employers to invest in inclusive practices and their opinion on these practices, directly influences whether labour market inclusion is achieved [34]. Research shows that the share of employers engaging in inclusive behaviour remains low due to various barriers they experience. For instance, research shows that employers may be reluctant to hire vulnerable workers due to lacking knowledge about how to facilitate these workers, biased expectations on their productivity, safety concerns, or negative attitudes of customers or co-workers [35–39]. Therefore, it is important to shed more light on organisational practices that employers themselves find relevant to include vulnerable workers [40, 41].

The primary responsibility of practices aimed at sustainable inclusion of vulnerable workers lies within the Human Resource (HR) department of an organisation, as HR is thought to have the primary responsibility for addressing social issues, such as inclusion, which were initially externalised to the sphere of public policy [3, p. 4]. Importantly, HR is not a stand-alone instrument. Rather, it should be aligned with other organisational practices and departments, such as facility management, e.g., by making workplaces accessible for vulnerable workers [3, 38]. Therefore, this scoping review is not solely focused on HR, but studies organisational policies and practices aimed at inclusion more broadly. Together and in alignment, these practices are thought to be the most supportive of workplace inclusion [42].

To gain insight in employers’ perceptions, this scoping review shifts the focus from studies based on employee samples to studies based on employer samples. Our research question is: ‘What organisational policies and practices do employers report to apply and find valuable for the inclusion of disabled persons, persons with a migration background, long-term unemployed persons, and low-educated persons?’. The insights resulting from our scoping review may support employers in the selection and application of relevant practices. Besides, this study provides the opportunity to systematically compare insights regarding key practices based on the perceptions of vulnerable workers versus those based on the perceptions of employers. Moreover, by including four specific vulnerable groups within the scope of our review, we can study the extent to which employer perspectives of different inclusive HR practices have been covered in the existing literature in relation to each of these groups and we can identify research gaps.

## Method

### Selection Criteria

To systematically map the research on organisational practices aimed at inclusion of vulnerable groups, from employers’ perceptions, a scoping review of qualitative and quantitative empirical research articles was conducted according to the PRISMA-ScR checklist [43]. Articles were included if they (1) studied the importance or application of (HR) policies or practices aimed at inclusion of vulnerable groups either quantitatively or qualitatively (2) were based on the perceptions of an organisational representative, meaning that the sample of the study consisted of representatives of the organisations (e.g., HR managers, CEOs, CHROs, presidents, supervisors, or directors). Furthermore, articles were included if they (3) studied the application or importance of these practices aimed at one (or multiple) of the following vulnerable groups: migrants, disabled workers, low-educated workers, or long-term unemployed workers. These categories were broadly operationalised, to capture a wide range of papers for each subgroup. In addition, studies were included if they (4) were published between 2000 and April 1st, 2022, (5) in international, peer-reviewed journals, as this helps to ensure the relevance and quality of the studies, and (6) were written in English. In the selection process of relevant articles, we applied the criteria above in numerical order.

### Search Strategy

As a first step in determining adequate search terms, we conducted an initial search for key articles in rehabilitation literature, in order to determine recurring terminology. This list of recurring key terms was refined by all authors, resulting in three groups of search terms, which each strive to capture the essence of this review: finding practices *aimed at the inclusion* of *migrants, disabled workers, low-educated workers, or long-term unemployed workers* that *employers* value. In order to meet the first selection criterion, “rehabilitation”, “inclusion”, “include”, “vocational opportunity”, “reintegration”, “disability management” or “activation” were used. To address the second selection criterion, the terms “HR”, “organisations”, “organisation”, “company”, “manager”, “management” or “employer” were used. To meet the third selection criterion, the search terms “migrant”, “disability”, “disabled”, “low-educated” or “unemployed” were included. The three groups of search terms were combined with the Boolean operator AND, the search terms within these three groups were combined with the operator OR. A full list of the search terms, relating to the three selection criteria, can be found in Online Appendix 1.

**Fig. 1:**
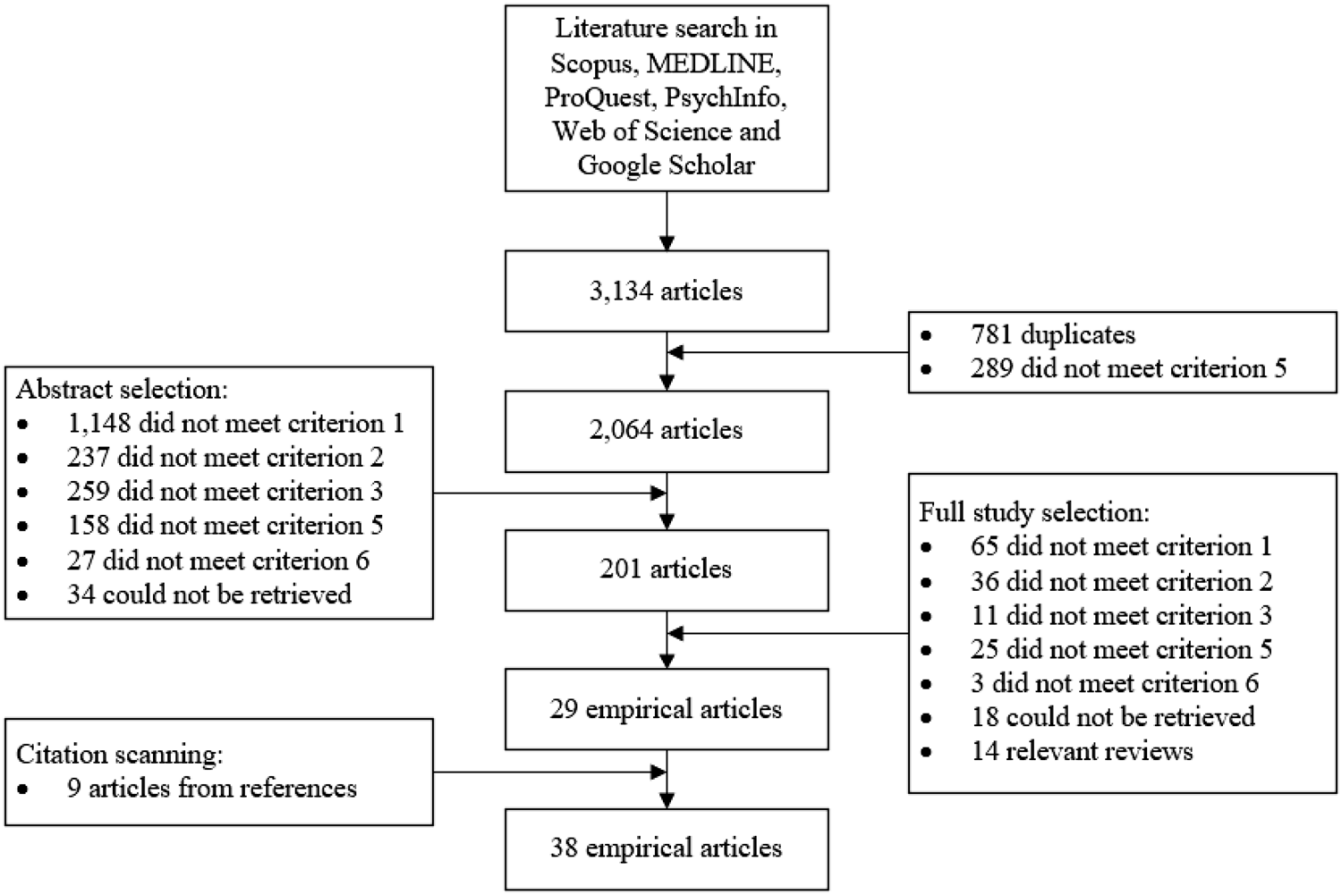
Scoping literature search and selection

The search terms were entered into six electronic databases in April 2022: MEDLINE, Scopus, ProQuest, PsychInfo, Google Scholar and Web of Science. This initial search resulted in 105 hits in MEDLINE, 273 hits in Scopus, 763 hits in ProQuest, 395 hits in PsychInfo, 744 hits in Google Scholar and 854 hits in Web of Science (total of 3,134 hits). Of these 3.134 hits, 781 were duplicates and 289 were not published in scientific journals, resulting in 2.064 unique journal articles. The titles and abstracts of these articles were scanned according to the numerical order of six selection criteria and 201 articles were selected. The main reason for exclusion was that 1.148 articles did not describe organisational policies or practices (criterion 1), but for example pain management interventions in 73 articles, weight management in 59 articles and medication management in 52 articles. In addition, 259 articles were excluded which did study (either qualitatively or quantitatively) inclusion practices from the perspective of a sample of organisational representatives, but aimed at a different target group, such as students in 49 articles, or medical patients (without disabilities) in 43 articles (criterion 3). The 201 remaining articles were examined in detail by reading the full-text version of the articles, after which 29 articles were included. The main reasons for exclusion were a lack of organisational policies or practices in the study, but rather general notions on organisational inclusion (criterion 1) in 65 studies, or a lack of organisational actors used as a sample, but for instance an employee sample (criterion 2) in 36 studies. Besides these empirical articles, 14 relevant reviews were identified in the process of full-text selection e.g., [42, 44–52]. These reviews were not included in the final sample, but the reference lists were scanned to retrieve additional empirical articles. This resulted in an additional nine studies that met all selection criteria. These articles were not retrieved with the aforementioned search terms, because of (1) the use of the term ‘diversity’ instead of ‘inclusion’, (2) no mentioning of specific vulnerable workers or (3) the mentioning of specific practices in the title, rather than general terms such’practice’. Figure 1 displays the selection process.

### Analysis Strategy

The authors scanned each article on the following information: (1) author(s); (2) publication date; (3) vulnerable group the policy or practice addressed; (4) respondents’ job within the organisation; (5) sample size; (6) country; (7) study design; (8) policy or practice for inclusion; (9) outcome measure; and (10) limitations and entered this coded information in separate spreadsheet for quantitative, qualitative and mixed methods studies. Next, the seperate policies or practices described in the 38 articles were entered into a new spreadsheet and labeled by the first author, resulting in a detailed overview of all relevant practices mentioned in the final sample. This resulted in an overview of 251 (partially overlapping) practices described in the final sample. Subsequently, all four authors coded all practices into clusters. The clusters were discussed until inter-rater agreement was reached. In this sense, reliability and interpretive validity of this scoping review and the analysis of practices were ensured by (1) applying a structured strategy and template in the literature search and data extraction, and (2) shared coding and categorizing of the practices with all four authors.

### Quality Assessment

To assess the quality of the quantitative, qualitative and mixed methods studies that were included in the final sample, the Standard Quality Assessment Criteria were applied [53]. This quality assessment tool can be used to assess the quality of articles with various designs, thereby allowing the assessor to estimate the relative strength of the included studies and to note any potential biases within these studies. The assessment criteria relate, amongst others, to assessments of the validity, methods, analyes, and reporting of the study. The 38 studies of the final sample were each assigned a score on the separate assessment criteria, as well as a total score based on the calculation provided by Kmet et al. [53]. Within this quality assessment, a total score of 1.0 indicates the highest possible quality and a total score of 0 indicates the lowest possible quality. The scores were discussed among the authors until inter-rater reliability was achieved.

## Results

The 38 articles that were included in the review applied different methodologies: 18 articles described quantitative studies [54–71], 16 articles described qualitative studies [72–87] and four studies applied mixed methods [88–91]. An overview of the articles, structured by methodology and listed from highest quality to lowest quality, can be found in Tables 1, 2 and 3.

**Table 1:** Relevant quantitative studies on policies and practices for the inclusion of vulnerable workers, listed from highest quality to lowest quality

	Study ID	Vulnerable group	Sample	*N*	Country	Design	Policy/practice	Results
1	Elkhwesky et al. (2021)	People with disabilities	HR managers and employees of hotels	1146	Egypt	Cross-sectional survey	Providing an equal treatment for disabled employees Hiring a number of disabled individuals in the F & B department according to the Egyptian governmental law (5%) Providing equal recruitment and advancement opportunities for disabled employees Providing a full payment for ill employees during an ill leave Providing equal and appropriate training opportunities for disabled employees Informing newly disabled employees with job instructions and rules, and health and safety procedures followed in the workplace Providing a required awareness training about rights and needs of disabled personnel in the workplace Fairness in the performance evaluation of disabled and non-disabled employees Providing suitable entrances, toilets, and washrooms for disabled employees Providing a proper working time for disabled employees Providing the opportunity to return to the work after absence due to injury or illness Providing suitable, safe, and healthy work environment for disabled employees Providing fair benefits, such as transport and housing for disabled employees	*Mean importance as disability management practice (M1; 1–5), mean level of implementation (M2; 1–5) and the significance of the gap between the two* *M1* = 4.40; *M2* = 3.77; *p* < 0.001 *M1* = 4.18; *M2* = 3.48; *p* < 0.001 *M1* = 4.22; *M2* = 3.49; *p* < 0.001 *M1* = 4.38; *M2* = 3.57; *p* < 0.001 *M1* = 4.36; *M2* = 3.51; *p* < 0.001 *M1* = 4.42; *M2* = 3.66; *p* < 0.001 *M1* = 4.36; *M2* = 3.57; *p* < 0.001 *M1* = 4.37; *M2* = 3.75; *p* < 0.001 *M1* = 4.33; *M2* = 3.36; *p* < 0.001 *M1* = 4.28; *M2* = 3.49; *p* < 0.001 *M1* = 4.33; *M2* = 3.65; *p* < 0.001 *M1* = 4.40; *M2* = 3.73; *p* < 0.001 *M1* = 4.21; *M2* = 3.32; *p* < 0.001
2	Luu (2018)	People with disabilities	Supervisors in IT	193	Vietnam	Longitudinal questionnaires	Disability inclusive HR practices	*Association with work engagement* *β* = 0.31, *p* < 0.01
3	Maini & Heera (2019)	People with disabilities	(HR) managers, general managers, senior managers	108	India	Cross-sectional, Questionnaires	Inclusive culture Disability-HRM fit Top management commitment Supportive workplace culture	*Odds ratio for likelihood to include people with disabilities* Odds ratio = 2.083, *p* = 0.000 Odds ratio = 1.167, n.s. (*p* = 0.055) Odds ratio = 0.628, *p* = 0.000 Odds ratio = 0.608, n.s. (*p* = 0.057)
4	Moore et al. (2010)	People with disabilities	Senior managers	190	USA	Cross-sectional, questionnaires	Top management support Top management vision Supportive practices	*Parameter estimates in model* **Top management support → practices***:* *b* = 0.40, *p* = 0.05 **Top management vision → practices***:* *b* = 0.540, *p* = 0.05 **Practices → Managers with disabilities**: *b* = 0.15, *p* = 0.04 **Support x Vision → practices**: *b* = 0.33, *p* = 0.05
5	Pérez-Conesa et al. (2020)	People with disabilities	Human Resource Managers	46	Spain	Cross-sectional, questionnaires	Strategic plan to normalise disability Collaboration with the local community Development of strategic alliances Employees feedback in internal survey Adaptation of communication plan Defining commitment and goals for inclusion	*Parameter estimates in model* **Strategic plan → inclusion**: *V* = .364, *p* = 0.018 **Community → recruitment**: *V* = .506, *p* = 0.001 **Alliances → recruitment:** *V* = .357, *p* = 0.024 **Feedback surveys → communication**: *V* = .430, *p* = 0.013 **Communication plan → recruitment**: *V* = .453, *p* = 0.004 **Define commitment → training**: *V* = .361, *p* = 0.019
6	Chan et al. (2020)	People with disabilities	Employers in charge of hiring	466	USA	Cross-sectional, online surveys	Include disability in policies Statement of commitment to hiring Have a stay-at-work retention policy Include disability in diversity training Include disability in employee orientation Recruitment strategies for people with disabilities Internship and summer employment programs Participate in job fairs for people with disabilities Have in-house disability management personnel Senior leadership communicates commitment Identify and select partners for recruitment Internal and external resources for support Annual targets to assess employment goals Senior executive with a disability Hiring manager with a disability Emergency preparedness policy Report inclusion progress to senior management Communicate emergency preparedness policy Have process to assess website for compliance Offer employee assistance programs Offer health and wellness programs Offer health care coverage Offer short-term benefits Display non-discrimination language Accommodations in recruitment process Contact with employment agencies Disability accommodation policy Have an accommodations budget Have a mechanism to assess the number of people with disabilities Have a mentoring program Accessible workplace Have a form for self-identification of disability Senior leadership communicates commitment Provide disability inclusion training for recruiters	*Mean importance for inclusion (1–4)* *M* = 3.32 *M* = 3.21 *M* = 3.21 *M* = 3.19 *M* = 3.16 *M* = 3.05 *M* = 2.81 *M* = 2.91 *M* = 3.80 *M* = 3.09 *M* = 2.99 *M* = 3.08 *M* = 2.94 *M* = 2.11 *M* = 2.19 *M* = 3.23 *M* = 2.74 *M* = 3.09 *M* = 3.16 *M* = 3.27 *M* = 3.36 *M* = 3.62 *M* = 3.30 *M* = 3.42 *M* = 3.21 *M* = *2.71* *M* = 3.28 *M* = 2.96 *M* = 2.86 *M* = 3.02 *M* = 3.35 *M* = 3.23 *M* = 2.78 *M* = 3.04
7	Erickson et al. (2014)	People with disabilities	HR professionals	675	USA	Online and phone surveys	Include disabilities in diversity statement Require subcontractors to adhere to norms Community relationships Training on non-discrimination Awareness training for managers Awareness training for employees Accommodation officer Flexible leave arrangements Flexible arrangements for all employees	*% of subsample (n) that applies this* 58.8% (*n* = 609) 57.2% (*n* = 558) 53.9% (*n* = 622) 67.3% (*n* = 642) 64.6% (*n* = 638) 54.7% (*n* = 649) 74.1% (*n* = 610) 71.2% (*n* = 556) 56.5% (*n* = 612)
8	Chordiya (2020)	Federal employees with disabilities	representative without disability	1,647,091	USA	Longitudinal, Federal Employees Viewpoint Survey	Organisational fairness Openness Supportiveness Cooperativeness Empowerment	*Odds for lowering turnover* Odds ratio = 0.825, *p* < 0.001 Odds ratio = 1.05, not significant Odds ratio = 1.00, not significant Odds ratio = 0.987, p < 0.001 Odds ratio = 0.801, p < 0.001
9	Bezyak et al. (2020)	People with disabilities	Employers (managers, CEOs, HR managers, etc.)	180	USA	Cross-sectional, online survey	Collaborate with rehabilitation agency Internship program Trial employment program Including disability in policies Adapted interviewing process External recruitment agency	*Correlation intention of employers to hire disabled workers* *r* = .38 (*p* < 0.001) *r* = .37 (*p* < 0.001) *r* = .32 (*p* < 0.001) *r* = .48 (*p* < 0.001) *r* = .28 (*p* < 0.001) *r* = .31 (*p* < 0.001)
10	Dong et al. (2012)	People with disabilities	Employers of people with disabilities	164	USA	Cross-sectional questionnaire	Supervisor support Support in accommodations Easy-to-use accommodations Communication efforts Matching accommodations to job requirements Accommodation policies Supervisor involvement in accommodations Formalisation of accommodation process Perceived fairness of accommodations	*Importance as accommodations (1–5)* *M* = 4.20 *M* = 4.32 *M* = 3.80 *M* = 4.25 *M* = 4.25 *M* = 4.16 *M* = 4.04 *M* = *3.17* *M* = 2.77
11	Houtenville & Kalargyrou (2012)	People with disabilities	President, vice president, HR manager, director, Supervisor	320	USA	Cross-sectional, survey	Flexible work schedule Disability awareness training Training existing staff Visible top management commitment Mentoring Using a specialised recruitment source Short term on the job assistance Assistive technology	*% of subsample indicating this is helpful* 77.1% (*n* = 237) 76.9% (*n* = 237) 73.4% (*n* = 237) 72.4% (*n* = 237) 71.1% (*n* = 237) 69.0% (*n* = 237) 66.1% (*n* = 237) 63.8% (*n* = 237)
12	Salkever et al. (2000)	People with disabilities	Frontline managers	273	USA	Cross-sectional, questionnaires	Bundle of job accommodations	*% Of sample reporting to apply this* 87.18%
13	Habeck et al. (2010)	People with disabilities	Employers experienced in disability management	95	USA	Cross-sectional questionnaires	Clear and compelling mission Fair compensation Awareness of benefits and services Credible managers and equitable treatment Open communication by leaders Safe and attractive work environment Leadership and supervisor training Jobs and performance related to mission Involvement in decision-making Career advancement opportunities Flexible benefits package Mentoring and support Employee surveys Flexible work accommodations	*Importance as retention practice (1–5)* *M* = 4.34 *M* = 4.30 *M* = 4.29 *M* = 4.26 *M* = 4.16 *M* = 4.13 *M* = 4.12 *M* = 4.05 *M* = 3.99 *M* = 3.97 *M* = 3.89 *M* = 3.75 *M* = 3.60 *M* = 3.56
14	Solovieva et al. (2011)	People with disabilities	Employer who received a request for accommodation	233	USA	Cross-sectional, online survey	Buying equipment Changing the work schedule Modifying the worksite Modifying the equipment Working from home/telework Education for co-workers Reassigning to another job Providing interpreter, reader, job coach Changing workplace policy Providing information in an alternative format	*% Of sample reporting this as top priority* 21% 21% 12% 12% 8% 7% 5% 4% 4% 3%
15	Hartnett et al. (2011)	People with disabilities	Employers	387	USA	Cross-sectional, survey	Flexible work arrangements for all employees Changes in work schedule Buying new products or equipment Modifying the worksite	*% of sample that applies this practice* 56.5% 22.9% 12.7% 5.3%
16	Kaye, et al. (2011)	People with disabilities	HR managers and managers working with reluctant organisations	463	USA	2 questionnaires	Awareness training for supervisors and managers Accommodation officer Guidelines for disability issues System for accommodation requests External expertise source Diversity specialist Written policy of non-discrimination	*% of sample indicating this is helpful* 74.4% 66.8% 65.2% 65.2% 60.6% 58.4% 50.7%
17	Bonoli (2014)	Long-term unemployed	(HR) managers, owners, recruitment manager	535	Switzerland	Cross-sectional questionnaires	Temporary job placement Making use of trustworthy references On-the-job supervision	*% of sample indicating this as relevant* 41.8% 39.8% 10.2%
18	Winter et al. (2016)	People with disabilities	Health and Safety Managers	88	Canada	Cross-sectional, web-based survey	Modified work and modified hours Accessibility accommodations	*% Of sample reporting to apply this* 94% 78%

**Table 2:** Relevant qualitative studies on policies and practices for the inclusion of vulnerable workers, listed from highest quality to lowest quality

	Study ID	Vulnerable group	Sample	*N*	Country	Design	Policy/practice	Results
1	Fujimoto et al. (2014)	People with disabilities	Managers or government officials	40	Australia	Structured interviews	Accessibility accommodations (physical, informational, relational) Diversity Champions Listening to minority voices	These practices were identified both by interviewees and reports to be relevant for inclusion of people with disabilities
2	Meacham et al. (2017)	Workers with intellectual disabilities	HR managers, department managers and supervisors	22	Australia	Case study methodology, interviews and focus groups	Social interaction with supervisor and colleagues Buddy systems Altruistic motivations	These practices were found to be effective techniques to provide on-going support to people with disabilities
3	Moore et al. (2020)	People with disabilities	Team managers	31	USA	Interviews	Creating a learning culture Courageous humility Authentic relationships Focus on problem solving and consensus building	These practices were applied by managers in order to adapt inclusive leadership and include people with disabilities
4	Lindsay et al. (2019)	Young people with disabilities under 30 years	Employers, managers and/or human resource manager with experience with YWD	18	USA, Canada	Semi-structured interviews	Accommodation procedures Training Mentorship Addressing stigma Open communication	These practices were found to be relevant for creating an inclusive environment within organisations
5	Gold et al. (2012)	People with disabilities	Employers	11	USA	Focus groups	Require employees with ‘hidden disabilities’ to address the need for accommodations Relationship building based on mutual trust	These practices were applied by employers in order to negotiate, grant, implement and evaluate reasonable workplace accommodations
6	Hazelzet et al. (2021)	Low-educated workers	HR managers	5	Netherlands	Interviews and focus groups	Interventions focused on physical work conditions Interventions focused on psychological work conditions Interventions aimed at improving communication among employees and employers	These interventions were found to be applied to stimulate self-direction, mutual trust and engagement among those involved
7	Meacham et al. (2019)	Workers with intellectual disabilities	Managers and supervisors of WWID	8	Australia	Interviews and focus groups	Training Buddy system Open communication Social connections	These practices were found to be relevant for the integration of workers with intellectual disabilities within several hotels
8	Gould et al. (2021)	People with disabilities	Diversity and inclusion ‘champions’	12	USA	Semi-structured interviews	Creating momentum and establishing buy-in Identifying an inclusion champion Obtaining initial top-level support Establishing an ERG Joining a community of practice Participating in an internal audit Develop a self-identification campaign Enhancing accommodation and return to work processes Continuing support for ERGs Connecting multiple stakeholders from across the organization Hosting training and awareness events Participating in a community of practice Creating mechanisms for implementation Prioritizing activities Connecting disability inclusion activities with business objectives Strategic planning by leadership and employee groups Naming future goals Infusing elements of universal design Evaluating progress toward goals Participating in internal audits and using information to identify areas for growth Continuing efforts for disability specific talent pipelines Taking on leadership positions within communities of practice Increasing disability representation in the organization	These practices were formed the initial start of the process of disability inclusion in the workplace These practices were applied to maintain disability inclusion and support an inclusive workplace Culture These practices were applied as next steps to further enhance disability inclusion through future planning
9	Strindlund et al. (2019)	People with disabilities	CEO, manager, HR manager or consultants	27	Sweden	Semi-structured interviews	Supportive external connections	This practice was found to be a successful strategy to address both employee and employer needs in inclusion
10	Gröschl (2007)	People with disabilities	HR directors	42	Canada	Interviews	Recruitment strategy Awareness training	These practices were applied in hotels as a means of increasing the inclusion for people with disabilities
11	Heera et al. (2017)	People with disabilities	Annual reports	50	India	Qualitative content analysis	Training and development Accommodations Inclusive recruitment and selection Promotion based on merit and talent Empowerment programs	These practices were disclosed in annual reports of Nifty companies in India as strategies for inclusion of people with disabilities
12	Irvine & Lupart (2008)	People with disabilities	Employers	10	–	In-depth interview	Specialized equipment Job coach Peer modelling Collaborative meetings Flexibility and choice for people with disabilities Structured environment Encouragement (reinforcement and redirection)	These practices were named by organisations as both formal and informal strategies for enhancing the inclusion of people with disabilities within organisations
13	Ball et al. (2005)	People with disabilities	Corporate website	100	USA	Qualitative content analysis	Employee Resource Groups Special interest groups for cultural awareness Disability in mission statement	These practices were found to be applied among Fortune 500 companies to include people with disability
14	Fillary & Pernice (2006)	Workers with intellectual disabilities	Organizational representatives	8	New Zealand	Semi-structured interviews	Job design Social opportunities Inclusion in customs	These practices were found in organisations with high inclusion for people with intellectual disabilities
15	Soares (2018)	People with disabilities	HR managers or those responsible for HR-related tasks	9	Brazil	Visits, spontaneous verbalizations and semi-structured questionnaires	Recruitment through advertisements in local newspapers and radios, nearby the premises of the company and from recommendations Training programs	These practices were applied by HR professionals in order to attract and maintain people with disabilities. Still, the employees were often let go after the legally required amount of time, as there was difficulty matching the training to the employee
16	Van der Torre & Fenger (2014)	People with disabilities	Websites and interviews with management	8	Netherlands	Qualitative content analysis, interviews	Education and training In-work benefits Improving physical accessibility Employer networks	These practices were found to be applied in order to achieve inclusion of people with disabilities in non-sheltered work

**Table 3:** Relevant mixed-methods studies on policies and practices for the inclusion of vulnerable workers, listed from highest quality to lowest quality

	Study ID	Vulnerable group	Sample	*N*	Country	Design	Policy/practice	Results
1	Hagner et al. (2015)	People with disabilities	On-site employment specialists	53	–	20-h online training, questionnaires, interviews with subsample	Shared equipment, co-worker help, social relations, employee training, fixed work schedule, regular performance review, typical routine for pay distribution	Inclusion of people with disabilities
2	Ebuenyi et al. (2020)	People with mental disabilities	(Potential) employers from rural and urban employment settings	10 (int.) + 158 (quest.)	Kenya	Interviews and questionnaires within different samples	Close supervision at the workplace, flexibility in work schedule and workplace, adjusting job tasks, creating an open and accepting atmosphere	Inclusion of people with mental disabilities in the workplace
3	Bento al. (2018)	People with disabilities	HR managers, HR leader, consultant, diversity managers	12	Norway	Semi-structured interviews and the Norwegian Disabled People LFS data	Providing adaptations in response to policy measures, such as accessibility of the premises of both the organization and their clients Changes of work tasks Changes of working time Physical adaptations of the workplace	Making changes in working time showed a positive effect on the employment of people with disabilities
4	Currier et al. (2001)	People with disabilities	Recognised experts in disability management	44	USA, Canada	Semi-structured interviews and follow-up questionnaires	Monitoring the impact of disability management programs, training in best practices, building organizational capacity, promoting the program internally and externally, training externally and internally, corporate analysis of costs	Relative importance for effective disability management

The 38 studies were conducted in 14 different countries (Australia, Brazil, Canada, Egypt, India, Kenya, the Netherlands, New Zealand, Norway, Spain, Sweden, Switzerland, USA, and Vietnam) and sample sizes ranged from eight to 1.647.091 participants. Most of the studies (20) were conducted in the USA or Canada. A variety of organisational actors participated, such as HR managers, supervisors, CEOs, and directors. Most of the quantitative studies had a cross-sectional design with questionnaires, while the majority of the qualitative studies used interviews. The four mixed methods studies all combined questionnaires with interviews. 36 articles discussed practices aimed at workers with disabilities (e.g., physical, intellectual, or developmental disabilities), which aligns with previous research that shows that wider conceptualisations of vulnerable workers (e.g., including migrant workers) have received little to no attention in relation to the employer’s perspective [3]. The remaining two articles discussed long-term unemployed workers [55] and low-educated workers [86]. Even though explicit search terms were used for migrant, articles were not retrieved, even after additional searches. In total, 12 studies investigated the value of policies or practices, 18 studies investigated the application of policies or practices, one study focused on the discrepancy between perceived importance and application of practices, and seven studies investigated effects of policies or practices on inclusion.

### Quality Assessment and Risk of Bias

In order to assess the potential biases of the studies included in the final sample, the Standard Quality Assessment tool from Kmet et al. was applied [53]. The results of this assessment are presented in Online Appendix 2. The quality scores ranged from 0.70 to 0.95 for quantitative studies, from 0.60 to 0.90 for qualitative studies and from 75 to 0.90 (quantitative elements) and 0.60 to 0.80 (qualitative elements) for mixed methods studies. Among the quantitative studies, all studies were above the threshold of 0.70, indicating that they were all ‘good’ quality studies. Seventeen quantitative studies were even considered to have a ‘strong’ quality, with a score of above 0.80. For the qualitative articles, three articles scored below 0.70, indicating lower quality, and three articles were rated exactly at the threshold of sufficient quality (0.70). The most common limitation among our final sample of qualitative and quantitative studies was related to the study samples, which were often relatively small, based on convenience sampling, or did not include potentially relevant cases or settings, thereby limiting the representativeness of the results (27 studies). Another common limitation was a solely descriptive design (e.g., mean importance scores of practices, percentages of employers that applied a practice), without including any estimate of variance or causality (11 studies). Also, results were often based on cross-sectional data, lacking in robustness or were not based on validated scales (16 studies). For qualitative studies in particular, verification procedures and reflexivity of the researchers was often lacking (18 studies).

### Policies and Practices for Inclusion

We encountered seven types of practices regarding the inclusion of vulnerable workers that were described from employers’ perceptions as either valued or applied in the organisational context: senior management commitment (13 articles), recruitment and selection (8 articles), performance management and development practices (9 articles), job accommodations and redesign of work (22 articles), supportive culture (27 articles), external collaborations (7 articles), and monitoring (6 articles). An overview of the practices is presented in Table 4. In the paragraphs below, we discuss the findings regarding these practices.

**Table 4:** Summary of the policies and practices named in the literature

*Senior Management Commitment*
Inclusion of disability in the organization’s policies and mission statement
Strategic plan for normalizing disability
Policy of non-discrimination and openly addressing stigma against disability
Internal and external promotion of disability-inclusive programs
Involvement and commitment of (senior) management to inclusion with a vision
*Recruitment and Selection*
Inclusive recruitment and selection strategy
Collaboration with external parties in recruitment, such as vocational rehabilitation agencies
Internship programs for people with disabilities or participation in job fairs
Diverse recruitment team
Accommodations for in the recruitment process (e.g., different communication format)
Open communication in recruitment process
*Performance Management and Development Practices*
Disability-HRM fit with disability inclusive (performance management) practices
On-the-job training for people with disabilities
Career advancement opportunities based on merit for people with disabilities
Fair compensation and flexible benefits
Regular performance reviews
Wellness programs and healthcare support, specialised for people with disabilities
Include work and disability in all relevant HR policies
*Job Accommodations and Redesign of Work*
Flexible work schedules, locations and leave arrangements
Modified or partial work duties
Accessibility of the workplace
Adapted furniture or equipment
Accommodations officer and system for accommodations request
Budget reserved for accommodations
*Supportive Culture*
Inclusive culture (e.g., fairness, cooperativeness, empowerment, encouragement)
Inclusion in social opportunities and customs
Support in socialization
Management support (e.g., inclusive leadership, mentoring systems)
Co-worker support (e.g., buddy systems, peer modelling or employee resource groups)
Disability (awareness) training
*External Collaborations (excl. Recruitment)*
Strategic alliances with experts, other organisations, or vocational rehabilitation agencies
Employer networks for inspiration and visibility
Requirements for subcontractors or suppliers
*Monitoring* Annual targets for disability management and the amount of people with disabilities
Corporate analysis of costs related to disability management
Mechanism to assess the number of people with disabilities
Involvement of people with disabilities in decision-making
Employee surveys aimed at feedback from minorities

#### Senior Management Commitment

An important theme in the literature was senior management commitment, referring to practices related to the active role of senior management in attuning the organisation towards support for inclusion [65]. Six studies indicated that a clear vision on inclusion of senior management, as well as affirmative communication concerning the commitment was seen as highly important for the inclusion of persons with disabilities e.g., [56, 62, 85]. Maini and Heera [65] found that organisations with explicit senior management commitment were 0.63 times more likely to be inclusive compared to those that lack commitment. Organisations may use various practices to demonstrate management commitment. For instance, Pérez-Conesa et al. [67] found that defining an explicit statement on commitment related to inclusion of persons with disabilities was applied by 42.8% of their sample of personnel managers and was positively related to more advanced inclusion practices—such as diversity training that aims to develop awareness and sensitivity for diverse issues at work. Other examples were stating goals for the inclusion of persons with disabilities in the organisation’s mission statement [54, 60, 71], dedicating attention to disability in all organisational policies and procedures [59] or publishing a policy on non-discrimination [56]. Additionally, developing a strategic plan to normalise disability was found to be positively related to labour inclusion of persons with disabilities [67]. Lastly, moving beyond solely promoting senior management’s commitment, openly addressing stigma within the organisation was valued [63, 77].

#### Recruitment and Selection

Five studies indicated that an inclusive recruitment strategy was an important practice for inclusion of persons with disabilities [56, 71, 75, 76, 87]. Several practices for inclusive recruitment were described, such as participation in job fairs for persons with disabilities, trial employment programs, advertisements in local newspapers, or offering internship programs [54, 56, 85, 87]. Next to that, Bezyak et al. [54] found that collaboration with vocational rehabilitation agencies or external recruitment agencies to recruit persons with disabilities was positively correlated to inclusion. Internally, having a recruitment manager with a disability was found to be a helpful practice [54]. Within the recruitment process, it was found to be helpful to use interviewing processes that provide open communication [54, 79]. In addition, providing accommodations in the recruitment process was seen as relevant and entails “anything that is required, so they can be their most successful self at the interview”, such as providing a sign language interpreter or changing to a one-person interview [78, p. 17]. Lastly, Chan et al. [56] indicate that it was helpful if recruitment managers had to report progress on the recruitment of persons with disabilities towards a senior manager to increase accountability. Regarding long-term unemployed workers, Bonoli [55] found that employers valued recruitment of a long-term unemployed worker through a trusted reference or through a temporary job placement (e.g., an internship).

#### Performance Management and Development Practices

Next to recruitment, performance management and development practices were found to be positively related to the engagement of persons with disabilities [64]. Maini and Heera [65] argued that sound HRM practices aimed at an inclusive workplace entailed a broader spectrum of practices than solely recruitment and selection. Examples of these practices were fair compensation and advancement practices, regular performance reviews for workers with disabilities, a fair routine for pay distribution and a reward system with flexible benefits [56, 71, 76, 83, 90]. Next to this, performance management practices such as an understanding of the link between one’s individual performance and the organisational mission were highlighted [60]. Offering advancement opportunities, empowerment programs, and training or skill development opportunities were mentioned as important HRM practices to support the development of vulnerable workers [76, 79, 85, 87, 90]. Practices such as disability-inclusive emergency policies, wellness programs, work-family policies and health care coverage were proposed to achieve sustained well-being and development of employees with disabilities [56, 90].

#### Job Accommodations and Redesign of Work

The use of job accommodations was described as a continuing process, in which the right accommodations can address the needs of vulnerable workers in a fair way [58]. It was found that over 87% of organisations used at least one type of accommodations and that offering accommodations supported retention of persons with disabilities [66, 68]. Several practices were found to be relevant for offering necessary flexibility to workers with disabilities, such as flexible work schedules, locations, leave arrangements, modified work duties, breaks, light duty work, or shared tasks or shifts [59, 68–70, 73, 85, 86, 91]. Additionally, accessibility practices were indicated to be important accommodations [83]. Examples were adapted formats of communication, accessible elevators, washrooms, parking, handrails, ramps, transportation, technical aids at work, improved infrastructure, and adapted lighting [58, 61, 67, 69, 70, 74, 76, 78, 86, 88, 90, 91]. In line with this, adapted furniture or equipment was indicated to be important for safety and attractiveness of the workplace [60, 69, 71, 77]. Examples were wheelchairs, amplified telephone headsets, adjustable computer equipment, raised shelves or an interpreter or reader. Lastly, to encourage employees to indicate their need for accommodations the importance of accommodation management systems and accommodation officers were highlighted [59, 63, 68, 84]. Chan et al. [56] provide the example of an employee assistance program for accommodations with an assigned budget. In line with this, Dong et al. [58] indicate that easy-to-use accommodations systems, as well as supervisor involvement, were estimated as highly important.

#### Supportive Culture

Practices aimed at cultivating a supportive culture with supportive co-workers or supervisors was mentioned in 16 articles. Maini and Heera [65] found that organisations with an inclusive culture were 2.08 times more likely to include persons with disabilities, compared to those without an inclusive culture. Chordiya [57] found that practices aimed at organisational fairness, cooperativeness and empowerment significantly lowered turnover intentions among persons with disabilities. Openness and supportiveness, however, were not significantly related to turnover of persons with disabilities. Other supportive cultural practices were encouragement, positive reinforcement, redirection, a stimulating and accepting atmosphere with open communication, inclusion in customs and social opportunities, supported socialisation, demonstrating genuine care about the well-being of employees and credible and equitable treatment of persons with disabilities [65, 73, 77, 84, 86, 89, 90].

Other studies indicated the importance of practices aimed at supervisor support, mentoring and leadership based on relationship building, consensus building and a learning climate [56, 58, 60, 62, 78]. In particular, Moore et al. [81] indicated the importance of courageous humility of leadership, which “focuses on employee success rather than the traditional “doing it my way” approach.” (p. 99). With courageous humility, leaders demonstrate flexibility and willingness to adapt in order to meet the needs of the employees. Examples of supervisor support were stimulating inclusion in celebrations and socialisation by providing encouragement to get the employee started or providing the employee with relevant information [90].

Other practices were related to co-worker support, such as co-worker help, buddy systems, peer modelling, diversity champions, and employee resource groups [72, 74, 77, 80, 85, 90]. This involvement and support of colleagues stimulated fellowship and helped to identify barriers. In addition to the support that co-workers and supervisors may offer, job coaches offered assistance, supervision, and encouragement. One study noted, however, that job coaches should not engage in too much support, as that might impede independence at work [77].

In order to achieve organisation-wide support, 11 articles indicated the importance of disability (awareness) training for all employees to build organisational capacity e.g., [63, 69, 75, 78]. In addition, Chan et al. [56] highlighted the importance to embed disability in all trainings and to include disability training in employee orientation training and training for HR recruiters.

#### External Collaborations

External collaborations with experts on inclusion or disability management, other organisations, communities of practice, or rehabilitation agencies for support, expertise, or visibility were mentioned in six articles as helpful for inclusion of persons with disabilities [56, 59, 63, 82, 83, 85]. Pérez-Conesa et al. [67] indicated that strategic alliances with other organisations or partners in the community were positively related to inclusion. Additionally, Erickson et al. [59] found that requiring subcontractors or suppliers to adhere to non-discriminatory requirements enhances inclusion.

#### Monitoring

Monitoring the effects of practices was mentioned in five articles and was rated as highly essential by recognised experts in disability management [88]. Quantitative practices that helped to achieve this were annual targets that serve to evaluate employment goals, internal audits with goalsetting, measuring the number of persons with disabilities, or using a corporate analysis of the costs of disability management initiatives [56, 85, 88]. For more qualitative monitoring, involving employees in monitoring practices was indicated as important [60, 67, 74]. Interviews by Fujimoto et al. [74] indicated that monitoring by listening to minority voices is important in the monitoring and adaptation of inclusive practices. Pérez-Conesa et al. [67] found that asking feedback on disability management with surveys positively influenced internal communication on inclusion efforts.

### The Relation Between Policies and Practices and Inclusion

The results of the quality assessment and Tables 1, 2 and 3 showed that most studies in our sample did not analyse the relation between practices and policies and outcome variables. In seven higher quality quantitative studies, the relationship with outcome variables was addressed (Table 5) [54, 57, 64–67, 71]. Five studies reported solely statistically significant relations (i.e., parameter estimates, correlations, associations and mean differences) between organisational practices and policies for inclusion and various outcome measures, such as the recruitment of people with disabilities, the intentions of people with disabilities to leave the organization or the representation of people with disabilities in management positions [54, 64, 65, 67, 71]. In two studies non-significant statistical relations (i.e., odds ratio and parameter estimates) were reported as well [57, 66].

**Table 5:** Studies reporting a statistically significant relation between Organisational Policies and Practices for Inclusion and Outcome Measures

*Senior management commitment*		
Top management commitment	+	Likelihood to include people with disabilities (Maini and Heera 2019)
Top management support and vision	+	Representation of managers with disabilities, fully mediated by supportive practices to grow to leadership positions (Moore et al. 2010)
Strategic plan to normalise disabilities	+	Percentage of employees with disabilities (Pérez-Conesa et al. 2020)
Including disability in all organisational policies	+	Intention to hire people with disabilities (Bezyak et al. 2020)
*Recruitment and selection*		
Trial employment program	+	Intention to hire people with disabilities (Bezyak et al. 2020)
Internship program	+	Intention to hire people with disabilities (Bezyak et al. 2020)
Adapted interviewing process	+	Intention to hire people with disabilities (Bezyak et al. 2020)
Collaborate with external recruitment agency	+	Intention to hire people with disabilities (Bezyak et al. 2020)
*Performance management and development practices*		
Disability inclusive HR practices	+	Work engagement of disabled employees through a mediating effect on organisational identification (Luu 2018)
Supportive practices to grow to leadership positions	+	Representation of managers with disabilities (Moore et al. 2010)
Disability-HRM fit	*n.s*	Likelihood to include people with disabilities (Maini and Heera, 2019)
*Job accommodations and redesign of work*		
Adapting internal communication systems to employees with disabilities	+	Recruitment of people with disabilities (Pérez-Conesa et al. 2020)
*Supportive culture*		
Inclusive culture	+	Likelihood to include people with disabilities (Maini and Heera 2019)
Supportive culture	*n.s*	Likelihood to include people with disabilities (Maini and Heera 2019)
Moral leadership	+	Moderating effect on the relationship between inclusive HR practices and the organisational identification of disabled employees (Luu 2018)
I-deals with leader	+ +	Moderating effect on the relationship between inclusive HR practices and the work engagement of disabled employees (Luu 2018) Moderating effect on the relationship between organizational identification of disabled employees and their work engagement (Luu 2018)
Organisational fairness	−	Intention of employees with disabilities to leave the organisation (Chordiya 2020)
Cooperativeness	−	Intention of employees with disabilities to leave the organisation (Chordiya 2020)
Empowerment	−	Intention of employees with disabilities to leave the organisation (Chordiya 2020)
Openness	*n.s*	Intention of employees with disabilities to leave the organisation (Chordiya 2020)
Supportiveness	*n.s*	Intention of employees with disabilities to leave the organisation (Chordiya 2020)
*External collaborations*		
Strategic alliances	+	Recruitment of people with disabilities (Pérez-Conesa et al. 2020)
Collaboration with the local community	+	Recruitment of people with disabilities (Pérez-Conesa et al. 2020)
*Monitoring*		
Internal surveys to gather employee feedback	+	Adaptation of internal communication for people with disabilities (Pérez-Conesa et al. 2020)
Defining commitment and goals for inclusion	+	Internal training for inclusion (Pérez-Conesa et al. 2020)

+ indicates a positive, significant effect,− indicates a negative, significant effect, *n.s*. indicates a non-significant effect

## Conclusion and Discussion

In this scoping review, we mapped the literature on key organisational practices for the inclusion of vulnerable groups, as perceived by the employer. By doing so, we respond to the call for systematic attention to the employer’s perspective on these practices and identify relevant research gaps related to this call [56]. Our findings indicate that we can distinguish seven types of practices based on the perceptions of employers. These categories include senior management commitment, recruitment and selection, other HR practices, job accommodations and redesign of work, supportive culture, external collaborations and monitoring. These practices affect various stages of the employee journey, ranging from onboarding of the employee to advancement in the organisation [92], and hence, move beyond solely recruitment of vulnerable workers [40, 56, 93]. Furthermore, this scoping review identified a major gap in the literature, by pointing out that literature on the employer’s perspective insufficiently addresses the challenges and needs of migrant workers, long-term unemployed workers, and low-educated workers. Some of the specified practices for people with disabilities, such as training opportunities or redesign of work, may also address the needs of other groups like long-term unemployed workers, by providing growth opportunities and enhancing the accessibility of employment. Still, several challenges that are unique to other vulnerable groups, such as xenophobia, language barriers, or having no education, remain unaddressed in the current employer-focused literature.

We were able to identify practices and policies for the workplace inclusion that are widely applied and valued by employers, such as modifying work(places) or changing work schedules. Further,, we identified practices that are not yet widely applied but that are valued by employers, such as strategic plans for inclusion, sustainable employability training, mentoring systems, fair compensation and development opportunities, or evaluation of strategic goals. Additionally, the following practices stood out as having a positive impact on the employment and retention of people with disabilities: top management commitment and support, strategic plans to normalise disabilities, recruitment practices such as trial employment programs, internship programs, adapted interviewing processes and collaborations with external recruitment agencies, disability inclusive HR practices, supportive practices to grow to leadership positions, adapted internal communication systems, having an inclusive culture characterised by moral leadership, fairness, cooperativeness and empowerment, making i-deals with the supervisor, strategic alliances and collaborations with the local community, and lastly, using internal surveys and inclusion goal setting.

When comparing the practices that we identified based on employer perceptions to those found in previous employee-focused studies, similarities and additions were found. Firstly, we identified several similarities between the employee-focused literature and employer-focused literature. For instance, both types of studies highlight the importance of top management [26] and the importance of supportive relationships and accommodations [28]. This indicates that there are several promising practices that are key to increase inclusion of vulnerable groups according to both employees and employers. However, our findings also indicate some practices which are largely absent in the employee-focused literature, most notably the external collaborations between employers and monitoring practices. This is not surprising, as we can expect that these practices may not be directly perceived by the employee [29]. For instance, employees may not directly observe whether an organisation participates in networks on how to attract and retain vulnerable workers with other employers. Still, employers rate such practices as highly relevant for the visibility, support and continuous development of their inclusive practices. This finding stresses the importance of studying employer perceptions of relevant practices, as these perceptions are key to increasing the inclusion and labour market participation of vulnerable workers.

### Limitations and Future Research

Several critical remarks can be made with regard to this scoping review. Firstly, the scope of this study was limited to practices aimed at the inclusion of disabled workers, migrant workers, low-educated workers, and long-term unemployed workers. However, the results of the review showed that 36 of the 38 articles in the final sample addressed disabled workers. No articles were found on the inclusion of migrant workers, and only two articles discussed long-term unemployed or low-educated workers, even when performing additional searches with terms such as ‘foreign’, ‘immigrant’, ‘immigrant worker’, ‘migratory worker’, ‘labour migrant’, ‘uneducated’, ‘unqualified’, or ‘low-skilled’ (see Appendix Online 1). Despite the acknowledged vulnerability and poor working conditions of these groups, scant knowledge is available on the organisational practices that are beneficial for the inclusion of these groups, especially as discussed from the employer’s perspective [94–97]. Possible explanations for this are the lack of national policies regarding the inclusion of these groups in specific, as compared to policies regarding the inclusion of persons with disabilities. We therefore call for studies that focus on organisational practices aimed at the sustainable inclusion of migrant workers, long-term unemployed workers and low-educated workers.

Secondly, our review revealed that most of the studies included in the final sample predominantly showed results relating to the importance that employers attach to certain inclusive practices. This led to fairly high importance scores on most practices, as seen in the study by Habeck et al. [60], who showed importance scores ranging from 3.56 to 4.34 (on a scale from 1 to 5) for all 14 practices that were rated by employers. We recommend future research to include measures that capture the relative importance of organisational practices and policies for inclusion (e.g., by means of ranking). Still, focusing solely on importance ratings, may lead to a twisted image, since there is a significant difference between valuing and actually applying a practice [71]. This indicates that future studies should include measures that capture the application of practices.

In addition, although it is important to gain insight in the opinions of employers about the relevance of certain practices, it is also important to gain more insight in the effectiveness of these practices in terms of more objective criteria (e.g., the increase in the inclusion of vulnerable workers). In the current sample, only seven studies actually studied the relation between policies and practices and relevant inclusion outcome variables (e.g., number of people with disabilities hired, intention to hire people with disabilities, amount of people with disabilities in leadership positions). The design of these studies does not allow us to draw any conclusions on causality. In addition, the generalisability of the samples was often weak due to small samples within one specific organisation or sector. Together, these methodological limitations prevent us from drawing robust conclusions on the effects of the application of practices and policies aimed at vulnerable workers. Hence, the research field of the employer’s perspective would benefit greatly from more objective data instead of subjective indicators, and from designs that allow researchers to study causal effects of the application of practices, and testing such effects within representative samples of employers [57, 65, 67].

Next to this, even though this review has shown an extensive list of practices for inclusion, the exact content of these practices remains somewhat unclear. For instance, numerous studies emphasised the importance of disability awareness training for the organisation, without specifying what the exact content of such a training should be. Future research would benefit from further conceptualisation of inclusion practices. In extension, the perceived effectiveness of combinations of practices was often not explored. Except for Luu [64], most studies focus on single, but often somewhat overlapping, practices. Therefore, we suggest future research to explore the effects of bundles of (specified) practices, in order to estimate the effectiveness for inclusion of vulnerable workers.

Lastly, exclusively peer-reviewed articles written in English were included. This may have led to exclusion of policies or practices, discussed in different languages. For instance, the Inclusive Turnover Growth Intervention [De Nieuwe Banen Methodiek] is a Dutch method that focuses on anticipating the growth of an organisation and giving vulnerable workers an extended timeframe to prepare for such a future job. As it is currently only described in Dutch literature [98], this intervention was not included. Future research could focus on employer practices within different countries and in different languages, to compare these different insights.

### Practical Implications

In order to address the ongoing vulnerability of certain workers on the labour market in terms of job security, employee rights and pay, it has become abundantly clear that organisations must be recognised as key actors. Still, there is a lack of systematic attention in research to employer’s perceptions on relevant practices for the sustainable inclusion of vulnerable groups [34, 41]. Our findings may guide practitioners at all organisational levels to take an active role in achieving inclusion of vulnerable workers. For instance, we provide employers with insights on which accommodations for vulnerable groups can be offered within their organisations, what they can do to increase senior management commitment, and what monitoring practices may help to continuously develop and improve their inclusive organisation. These insights extend and enrich the already existing insights from the employee’s point of view. On the level of organisational leaders and senior management, our findings may stimulate and promote the realisation of inclusive climates, in which the seven types of practices for vulnerable workers are key. HR professionals are advised to develop and monitor inclusive HR (recruitment) practices, to make work accessible for all employees and to develop policies that enhance diversity at all organisational levels. To conclude, as Van Berkel [39] recently stated: “enthusiastic employers that share successful experiences with colleague employers are likely to be a crucial factor in increasing the number of motivated and participating organisations” (p. 13). Therefore, in line with our finding of the importance of collaborations between employers, we want to call on enthusiastic and experienced employers to inspire other organisations, researchers, experts, or rehabilitation agencies by sharing ideas, policies, or practices to enhance knowledge sharing of inclusive practices and modelling.

## Supplementary Information

Below is the link to the electronic supplementary material.

Supplementary file1 (DOCX 38 kb)

Supplementary file2 (DOCX 54 kb)

## Data Availability

Not applicable.
