# Correction: Organisational Policies and Practices for the Inclusion of Vulnerable Workers: A Scoping Review of the Employer’s Perspective

**DOI:** 10.1007/s10926-024-10242-7

**Published:** 2024-11-02

**Authors:** A. Kersten, M. van Woerkom, G. A. Geuskens, R. W. B. Blonk

**Affiliations:** 1 Department of Human Resource Studies, School of Social and Behavioral Sciences, Tilburg University, Warandelaan 2, 5037 AB Tilburg, The Netherlands; 2 Healthy Living, Netherlands Organisation for Applied Scientific Research (TNO), Leiden, The Netherlands; 3 Department of Psychology, Education and Child Studies, Center of Excellence for Positive Organisational Psychology, Erasmus University Rotterdam, Rotterdam, The Netherlands; 4 Optentia Research Focus Area, North-West University, Vanderbijlpark, South Africa

**Correction: Journal of Occupational Rehabilitation (2022) 33:245–266** 10.1007/s10926-022-10067-2

In this article, the reference citation numbering shifted and wrongly published.

In the subheading “Search Strategy” in second paragraph the date was incorrectly given as “September 2020” but should have been “April 2022”.

The original article has been corrected.

