# Supplementary material for: Organisational Policies and Practices for the Inclusion of Vulnerable Workers: A Scoping Review of the Employer’s Perspective
Source: J Occup Rehabil. 2022 Sep 9;33(2):245–66. doi: 10.1007/s10926-022-10067-2 (PMC9461424; doi:10.1007/s10926-022-10067-2)
Supplement: Supplementary file 1 — Supplementary file1 (DOCX 38 kb) [file 10926_2022_10067_MOESM1_ESM.docx]

# Appendix 1. Key Search Terms

| **Table 6.** Key Search Terms for the Scoping Review | |
| --- | --- |
| **Selection criterion 1**: Studies must be aimed at describing or testing the effects of (HR) policies or practices aimed at the inclusion of vulnerable workers | |
| “REHABILITATION”  “REHABILITATE”  “REHABILIT*”  “INCLUSION”  “INCLUSIVE”  “INCLUDE”  “INCLUS*”  “INCLUD*”  “DISABILITY MANAGEMENT”  “DISABILITY MANAG*” | “VOCATIONAL OPPORTUNITY”  “VOCATIONAL”  “REINTEGRATION”  “REINTEGRATE”  “REINEGRAT*”  “ACTIVATION”  “ACTIVATE”  “ACTIVAT*” |
| **Selection criterion 2**: Studies must use a sample of organisational representatives, meaning that the sample of the study consisted of representatives of the organisations (e.g., HR managers, CEOs, CHROs, presidents, supervisors, or directors) | |
| “ORGANISATIONS”  “ORGANISATION”  “ORGANISATION*”  “ORGANIZATIONS”  “ORGANIZATION”  “ORGANIZATION*” | “COMPANY”  “COMPANY*”  “MANAGER”  “MANAGEMENT”  “EMPLOYER”  “HR” |
| **Selection criterion 3:** Studies must explicitly describe practices aimed at one (or multiple) of the following vulnerable groups: migrants, disabled workers, low-educated workers, or long-term unemployed workers | |
| “MIGRANT”  “IMMIGRANT”  “IMMIGRANT WORKER”  “MIGRATORY WORKER”  “LABOUR MIGRANT”  “FOREIGN”  “DISABILITY”  “DISABLED”  “DISABIL*” | “LOW-EDUCATED”  “LOW EDUCATED”  “UNEDUCATED”  “UNQUALIFIED”  “LOW-SKILLED”  “UNEMPLOYED”  “LONG-TERM UNEMPLOYED” |

*Note*. The asterisk (*) is used as a Boolean modifier, to search for any article title including the stem of the word that precedes the asterisk. The search terms within different selection criteria were combined with the Boolean operator OR, and the different selection criteria were combined with the Boolean operator AND.
