# Supplementary material for: Organisational Policies and Practices for the Inclusion of Vulnerable Workers: A Scoping Review of the Employer’s Perspective
Source: J Occup Rehabil. 2022 Sep 9;33(2):245–66. doi: 10.1007/s10926-022-10067-2 (PMC9461424; doi:10.1007/s10926-022-10067-2)
Supplement: Supplementary file 2 — Supplementary file2 (DOCX 54 kb) [file 10926_2022_10067_MOESM2_ESM.docx]

# Appendix 2. Standard Quality Assessment of Final Sample

| **Table 7.** Standard Quality Assessment of quantitative and mixed methods studies | | | | | | | | | | | | | | | |
| --- | --- | --- | --- | --- | --- | --- | --- | --- | --- | --- | --- | --- | --- | --- | --- |
| **Quantitative papers** | | | | | | | | | | | | | | | |
| **Reference** | **1** | **2** | **3** | **4** | **5** | **6** | **7** | **8** | **9** | **10** | **11** | **12** | **13** | **14** | **Total score** |
| Bonoli 2014 | 2 | 1 | 1 | 2 | n/a | n/a | n/a | 1 | 2 | 1 | 1 | n/a | 1 | 2 | 14/20 = .70 |
| Bezyak et al. 2020 | 2 | 2 | 2 | 1 | n/a | n/a | n/a | 2 | 1 | 2 | 1 | n/a | 2 | 2 | 17/20 = .85 |
| Chan et al. 2020 | 2 | 1 | 2 | 2 | n/a | n/a | n/a | 1 | 2 | 2 | 2 | n/a | 2 | 2 | 18/20 = .90 |
| Chordiya 2020 | 2 | 2 | 2 | 2 | n/a | n/a | n/a | 1 | 2 | 1 | 1 | n/a | 2 | 2 | 17/20 = .85 |
| Dong et al. 2012 | 2 | 1 | 2 | 1 | n/a | n/a | n/a | 2 | 1 | 2 | 2 | n/a | 2 | 2 | 17/20 = .85 |
| Elkhwesky et al. 2021 | 2 | 2 | 2 | 2 | n/a | n/a | n/a | 2 | 2 | 2 | 1 | n/a | 2 | 2 | 19/20 = .95 |
| Erickson et al. 2014 | 2 | 2 | 2 | 2 | n/a | n/a | n/a | 1 | 2 | 2 | 1 | n/a | 2 | 2 | 18/20 = .90 |
| Habeck et al. 2010 | 2 | 1 | 2 | 2 | n/a | n/a | n/a | 1 | 1 | 2 | 1 | n/a | 2 | 2 | 16/20 = .80 |
| Hartnett et al. 2011 | 2 | 2 | 2 | 2 | n/a | n/a | n/a | 1 | 2 | 1 | 0 | n/a | 1 | 2 | 15/20 = .75 |
| Houtenville et al. 2012 | 2 | 2 | 2 | 2 | n/a | n/a | n/a | 1 | 2 | 1 | 1 | n/a | 2 | 2 | 17/20 = .85 |
| Kaye et al. 2011 | 2 | 2 | 2 | 1 | n/a | n/a | n/a | 1 | 2 | 1 | 0 | n/a | 1 | 2 | 14/20 = .70 |
| Luu 2018 | 2 | 2 | 2 | 2 | n/a | n/a | n/a | 1 | 1 | 2 | 2 | n/a | 2 | 2 | 18/20 = .90 |
| Maini et al. 2019 | 2 | 2 | 1 | 2 | n/a | n/a | n/a | 2 | 1 | 2 | 2 | n/a | 2 | 2 | 18/20 = .90 |
| Moore et al. 2010 | 2 | 2 | 2 | 1 | n/a | n/a | n/a | 2 | 1 | 2 | 2 | n/a | 2 | 2 | 18/20 = .90 |
| Pérez-Conesa et al. 2020 | 2 | 2 | 2 | 1 | n/a | n/a | n/a | 2 | 1 | 2 | 2 | n/a | 2 | 2 | 18/20 = .90 |
| Salkever et al. 2000 | 2 | 2 | 2 | 2 | n/a | n/a | n/a | 1 | 2 | 1 | 1 | n/a | 2 | 2 | 17/20 = .85 |
| Solovieva et al. 2011 | 2 | 2 | 2 | 2 | n/a | n/a | n/a | 1 | 2 | 1 | 1 | n/a | 1 | 2 | 16/20 = .80 |
| Winter et al. 2016 | 1 | 2 | 2 | 2 | n/a | n/a | n/a | 1 | 1 | 1 | 1 | n/a | 1 | 2 | 14/20 = .70 |
| **Mixed methods papers** | | | | | | | | | | | | | | | |
| **Reference** | **1** | **2** | **3** | **4** | **5** | **6** | **7** | **8** | **9** | **10** | **11** | **12** | **13** | **14** | **Total score** |
| Currier et al. 2001 | 2 | 2 | 2 | 2 | n/a | n/a | n/a | 1 | 1 | 1 | 1 | n/a | 2 | 2 | 16/20 = .80 |
| Ebuenyi et al. 2020 | 2 | 2 | 2 | 2 | n/a | n/a | n/a | 1 | 1 | 2 | 2 | n/a | 2 | 2 | 18/20 = .90 |
| Hagner et al. 2015 | 2 | 2 | 2 | 2 | n/a | n/a | n/a | 1 | 1 | 1 | 0 | n/a | 2 | 2 | 15/20 = .75 |
| Bento et al. 2018 | 2 | 2 | 2 | 2 | n/a | n/a | n/a | 1 | 1 | 1 | 2 | n/a | 1 | 2 | 16/20 = .80 |
| *Note*. The quality assessment of these papers was performed according to the Standard Quality Assessment Criteria of: Kmet, L.M., R.C. Lee, and L.S. Cook, *Standard quality assessment criteria for evaluating primary research papers from a variety of fields*. Vol. 22. 2004: Alberta Heritage Foundation for Medical Research Edmonton. | | | | | | | | | | | | | | | |

| **Table 8.** Standard Quality Assessment of qualitative and mixed methods studies | | | | | | | | | | | |
| --- | --- | --- | --- | --- | --- | --- | --- | --- | --- | --- | --- |
| **Qualitative papers** | | | | | | | | | | | |
| **Reference** | **1** | **2** | **3** | **4** | **5** | **6** | **7** | **8** | **9** | **10** | **Total score** |
| Ball et al. 20115 | 2 | 1 | 2 | 1 | 2 | 1 | 2 | 1 | 2 | 0 | 14/20 = .70 |
| Fillary et al. 2006 | 2 | 1 | 2 | 0 | 1 | 2 | 1 | 1 | 2 | 2 | 14/20 = .70 |
| Fujimoto et al. 2014 | 2 | 2 | 2 | 2 | 1 | 2 | 2 | 2 | 2 | 1 | 18/20 = .90 |
| Gold et al. 2012 | 2 | 2 | 2 | 1 | 1 | 2 | 2 | 2 | 2 | 1 | 17/20 = .85 |
| Gould et al. 2021 | 2 | 2 | 2 | 2 | 1 | 2 | 2 | 1 | 2 | 0 | 16/20 = .80 |
| Gröschl 2007 | 2 | 2 | 2 | 2 | 1 | 1 | 2 | 1 | 2 | 0 | 15/20 = .75 |
| Hazelzet et al. 2021 | 2 | 2 | 2 | 2 | 1 | 2 | 1 | 1 | 2 | 1 | 16/20 = .80 |
| Heera et al. 2017 | 2 | 2 | 2 | 2 | 1 | 1 | 2 | 1 | 2 | 0 | 15/20 = .75 |
| Irvine et al. 2008 | 2 | 2 | 1 | 1 | 1 | 1 | 2 | 1 | 2 | 2 | 15/20 = .75 |
| Lindsay et al. 2019 | 2 | 2 | 2 | 1 | 1 | 2 | 2 | 2 | 2 | 1 | 17/20 = .85 |
| Meacham et al. 2017 | 2 | 2 | 2 | 2 | 1 | 2 | 2 | 2 | 2 | 1 | 18/20 = .90 |
| Meacham et al. 2019 | 2 | 2 | 2 | 2 | 1 | 2 | 2 | 1 | 2 | 0 | 16/20 = .80 |
| Moore et al. 2020 | 2 | 2 | 2 | 2 | 1 | 2 | 2 | 2 | 2 | 1 | 18/20 = .90 |
| Soares 2018 | 2 | 2 | 2 | 1 | 1 | 2 | 1 | 1 | 2 | 0 | 14/20 = .70 |
| Strindlund et al. 2019 | 2 | 2 | 2 | 1 | 1 | 2 | 2 | 1 | 2 | 1 | 16/20 = .80 |
| Van der Torre et al. 2014 | 2 | 2 | 2 | 2 | 1 | 0 | 1 | 0 | 1 | 1 | 12/20 = .60 |
| **Mixed Methods papers** | | | | | | | | | | | |
| **Reference** | **1** | **2** | **3** | **4** | **5** | **6** | **7** | **8** | **9** | **10** | **Total score** |
| Currier et al. 2001 | 2 | 2 | 1 | 1 | 1 | 2 | 1 | 1 | 2 | 0 | 13/20 = .65 |
| Ebuenyi et al. 2020 | 2 | 2 | 1 | 1 | 1 | 2 | 1 | 0 | 2 | 0 | 12/20 = .60 |
| Hagner et al. 2015 | 2 | 2 | 1 | 1 | 1 | 2 | 2 | 2 | 2 | 1 | 16/20 = .80 |
| Bento et al. 2018 | 2 | 2 | 2 | 1 | 1 | 2 | 1 | 2 | 2 | 0 | 15/20 = .75 |
| *Note*. The quality assessment of these papers was performed according to the Standard Quality Assessment Criteria of: Kmet, L.M., R.C. Lee, and L.S. Cook, *Standard quality assessment criteria for evaluating primary research papers from a variety of fields*. Vol. 22. 2004: Alberta Heritage Foundation for Medical Research Edmonton. | | | | | | | | | | | |
